# IgG4-Related Retroperitoneal Fibrosis Presenting as Obstructive Acute Kidney Injury: A Case Report

**DOI:** 10.7759/cureus.107379

**Published:** 2026-04-20

**Authors:** M. Zaid Jarai, Sara Y Haider, Salma Eldesouki, Seyed Hashemi, Sima Nejad, Faisal Elbadawi, Elham Mahjoor Azad, Fariborz Bagheri

**Affiliations:** 1 Urology, Dubai Health, Dubai, ARE; 2 Internal Medicine, Dubai Health, Dubai, ARE; 3 Nephrology, Dubai Health, Dubai, ARE; 4 Rheumatology, Dubai Health, Dubai, ARE; 5 Family Medicine, Medcare Hospital, Dubai, ARE; 6 Urology, Dubai Hospital, Dubai, ARE

**Keywords:** acute kidney injury, igg4 -related disease, obstructive uropathy, retroperitoneal fibrosis, rituximab

## Abstract

Immunoglobulin G4-related disease (IgG4-RD) is a systemic fibroinflammatory disorder with retroperitoneal fibrosis (RPF) representing a recognized manifestation. Ureteric encasement by fibroinflammatory tissue may result in obstructive uropathy and acute kidney injury (AKI). We report the case of a 46-year-old male who presented with bilateral flank pain and severe AKI. Cross-sectional imaging demonstrated a retroperitoneal soft-tissue mass causing bilateral hydroureteronephrosis. Initial management with bilateral ureteric stent insertion resulted in marked improvement in renal function. Subsequent CT-guided biopsy revealed a fibroinflammatory spindle cell lesion consistent with IgG4-related RPF. The patient later re-presented with recurrent anuria and deterioration in renal function, raising concern for disease relapse. Rheumatology assessment confirmed IgG4-RD; however, initiation of definitive immunosuppressive therapy was deferred due to a concurrent urinary tract infection. Following resolution of infection, immunosuppressive treatment was commenced. This case underscores the diagnostic and therapeutic challenges of IgG4-related RPF and highlights the importance of multidisciplinary management, particularly in differentiating disease activity from superimposed infection.

## Introduction

Retroperitoneal fibrosis (RPF) is an uncommon fibroinflammatory condition characterized by the development of dense fibrotic tissue within the retroperitoneum, with potential encasement of adjacent structures, including the aorta, inferior vena cava, and ureters [1,2]. The etiology of RPF is diverse, historically classified into primary (idiopathic) and secondary forms; secondary RPF may be triggered by various factors, including malignancies (e.g., lymphoma), chronic infections (e.g., tuberculosis), prior radiation therapy, or certain medications such as methysergide and beta-blockers [1]. While these secondary causes must be systematically excluded, an increasing proportion of cases previously deemed idiopathic are now recognized as manifestations of immunoglobulin G4-related disease (IgG4-RD) [3,4]. IgG4-RD is a systemic immune-mediated fibroinflammatory disorder that can involve multiple organs, most commonly the pancreas, salivary glands, and kidneys [5].

Establishing the diagnosis of IgG4-related RPF can be challenging and typically relies on an integrated assessment of clinical features, radiological findings, histopathology, and supportive serological markers, including elevated serum IgG4 levels [6,7]. Ureteric involvement with subsequent obstructive uropathy is a frequent and clinically significant manifestation and may result in acute kidney injury (AKI) if not promptly recognized and managed [8,9]. Early decompression with ureteric stenting or percutaneous nephrostomy is therefore essential to preserve renal function [9,10].

Immunosuppressive therapy remains the cornerstone of treatment for IgG4-RD, with corticosteroids demonstrating high efficacy in inducing disease remission [11,12]. In patients with relapsing disease or steroid intolerance or resistance, B-cell-depleting therapy such as rituximab represents an effective alternative [13,14].

We report the case of a patient with IgG4-related RPF who presented with severe AKI secondary to bilateral obstructive uropathy. This case illustrates the complex interaction between mechanical obstruction and systemic inflammation, further complicated by a concurrent infectious process that delayed the initiation of definitive immunosuppressive therapy. It underscores the need for careful clinical judgment and a multidisciplinary approach in the management of IgG4-related RPF.

## Case presentation

A 46-year-old male from Jordan presented to the Emergency Department on 12 July 2025 with bilateral flank pain, vomiting, and dysuria. His medical history was notable for a documented penicillin allergy. He also reported an unintentional weight loss of approximately 8 kg over the preceding week.

**Figure 1:**
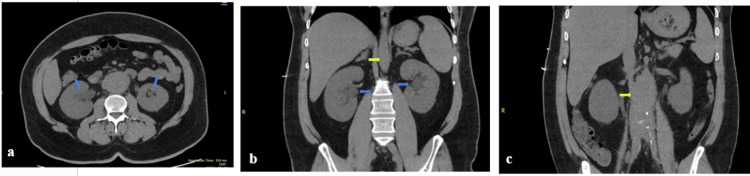
CT KUB demonstrating retroperitoneal fibrosis with bilateral hydronephrosis. Axial (a) and coronal (b and c) non-contrast CT KUB images demonstrating bilateral hydroureteronephrosis (blue arrows) with an associated lobulated retroperitoneal soft-tissue mass encasing the abdominal aorta, inferior vena cava, and bilateral ureters (yellow arrows), consistent with retroperitoneal fibrosis. CT KUB: Computed tomography of kidneys, ureters and bladder

Initial laboratory investigations demonstrated severe acute kidney injury, with a serum creatinine of 10.30 mg/dL on admission, rising to 11.00 mg/dL on 13 July 2025. Routine laboratory investigations are summarized in Table 1. A non-contrast computed tomography of kidneys, ureters, and bladder (CT KUB) performed on 12 July 2025 revealed a lobulated retroperitoneal soft-tissue mass encasing the abdominal aorta, inferior vena cava, and bilateral ureters, with associated bilateral hydroureteronephrosis (Figure 1). These findings were consistent with retroperitoneal fibrosis and correlated with the patient’s obstructive uropathy and renal failure.

**Table 1:** Baseline laboratory investigations at initial presentation. APTT: Activated Partial Thromboplastin Time, INR: International Normalized Ratio

Parameter	Value	Reference Range	Note
Vital signs			
Temperature ( degrees Celsius °C)	37	36.1 - 37.2 °C	
Blood pressure (millimeter mercury)	112/66	90 - 120/ 60 - 80 mmHg	
Heart rate (beat/minute)	76	60 - 100 bpm	
Respiratory rate (breath/minute)	15	12 - 20 breaths/ min	
Oxygen saturation (%)	99	≥95%	On room air
Anthropometric Measurements			
Weight (kilograms)	114		
Height (centimeters)	174		
Body mass index (kilograms/meter^2^)	37.7	18.5 - 24.9 kg/m^2^	Obese class II
Laboratory investigations			
Hemoglobin (g/dL)	10.8	13.0 - 17.0 g/dL	
White blood cells (10^3^/uL)	5.7	3.6 - 11.0 10^3^/uL	
Platelets (10^9^/uL)	300	150 - 400 10^9^/uL	
Sodium (mmol/L)	140	136 - 145 mmol/L	
Potassium (mmol/L)	4	3.4 - 4.5 mmol/L	
Chloride (mmol/L)	105	98 - 108 mmol/L	
Bicarbonate (mmol/L)	23.1	20 - 28 mmol/L	
Urea (mg/dL)	45	12 - 40 mg/dL	
Calcium (mg/dL)	9	9 - 10.2 mg/dL	
Phosphate (mg/dL)	4	2.7 - 4.5 mg/dL	
Uric acid (mg/dL)	8.4	3.4 - 7.0 mg/dL	
Creatinine (mg/dL)	10.30	0.70 - 1.20 mg/dL	
eGFR (ml/min/1.73m^2^)	6.5	>60 ml/min/1.73m^2^	
Urine protein to creatinine ratio	401	<50 mg protein/ g creatinine	
C-reactive protein (mg/L)	72.6	<5.0 mg/L	
Procalcitonin (ng/ml)	0.22	<0.05 ng/ml	
Aspartate aminotransferase (U/L)	25	<40	
Alanine aminotransferase (U/L)	23	<40	
Alkaline phosphatase (U/L)	51	40 - 129	
Bilirubin (mmol/L)	4	<21	
Albumin (g/L)	41	35 - 50	
Prothrombin time (seconds)	10.9	9.7 - 11.8 seconds	
INR	1.05	0.8 - 1.1	
APTT (seconds)	32.9	25.1 - 37.7 seconds	
Anti-nuclear antibody	Negative	< 1/100 - Negative ≥ 1/100 - Positive	
Immunoglobulin G4 (g/L)	0.70	0.0392 - 0.864 g.L	
Anti-glomerular basement membrane antibodies (U/ml)	<2	Up to 7 U/ml	
Complement - C4 (g/L)	0.44	0.10 - 0.40 g/L	
Complement - C3 (g/L)	1.50	0.90 - 1.80 g/L	

**Figure 2:**
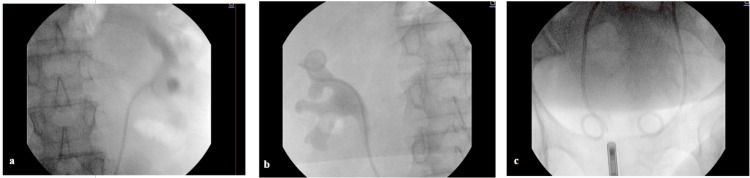
Fluoroscopic images following bilateral ureteric stent insertion. Intraoperative fluoroscopic images demonstrating successful bilateral ureteric stent placement, with appropriate positioning of the proximal coils within the renal pelves (a and b) and distal coils within the urinary bladder (c), achieving decompression of the obstructed upper urinary tracts.

**Figure 3:**
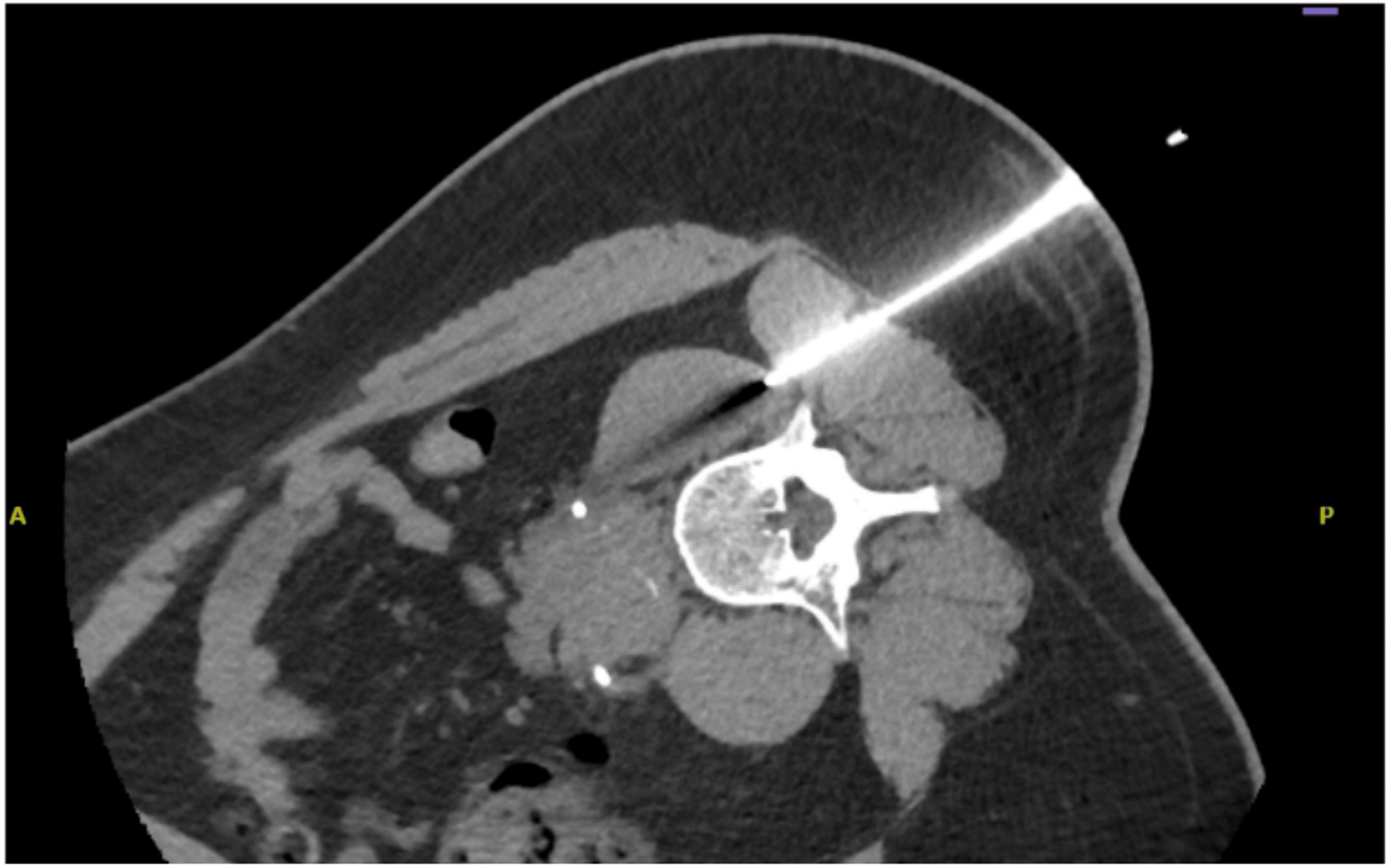
CT-guided biopsy of the retroperitoneal mass. Axial CT image demonstrating percutaneous CT-guided biopsy of the retroperitoneal periaortic soft-tissue mass, with the biopsy needle positioned within the lesion for tissue sampling. CT: Computed tomography

In view of the bilateral obstruction, the patient underwent bilateral ureteric stent insertion on 13 July 2025 (Figure 2). This resulted in a rapid and sustained improvement in renal function, with serum creatinine decreasing to 1.75 mg/dL by 28 July 2025. A CT-guided biopsy of the periaortic mass was performed on 15 July 2025 (Figure 3). Histopathological examination, reported on 25 July 2025, demonstrated a fibroinflammatory spindle cell lesion, with features suggestive of retroperitoneal fibrosis in the context of IgG4-related disease. Following stabilization of renal function, the patient was discharged with outpatient rheumatology follow-up.

The patient was re-admitted on 8 August 2025 with a one-day history of anuria, preceded by hematuria two days earlier. On admission, his serum creatinine had risen to 5.54 mg/dL and continued to deteriorate, peaking at 12.50 mg/dL on 12 August 2025. A repeat CT KUB performed on 8 August 2025 confirmed the previously identified retroperitoneal soft-tissue mass encasing the ureters, with bilateral ureteric stents in situ. However, persistent bilateral mild-to-moderate hydroureteronephrosis and perinephric fat stranding were noted, suggesting ongoing obstruction and inflammation despite stenting.

Given the rapid worsening of renal function, hemodialysis was initiated on 12 August 2025 following a nephrology consultation. After a multidisciplinary discussion between the nephrology and urology teams, bilateral percutaneous nephrostomy tubes were inserted on 14 August 2025 due to persistent hydronephrosis. Subsequent renal recovery was observed, with serum creatinine decreasing to 9.27 mg/dL on 14 August 2025, 5.46 mg/dL on 15 August 2025, 2.98 mg/dL on 16 August 2025, 2.36 mg/dL on 17 August 2025, and normalizing to 1.48 mg/dL by 25 August 2025. The marked improvement in urine output and renal indices following nephrostomy placement confirmed post-renal obstruction secondary to retroperitoneal fibrosis as the primary driver of his acute kidney injury.

**Figure 4:**
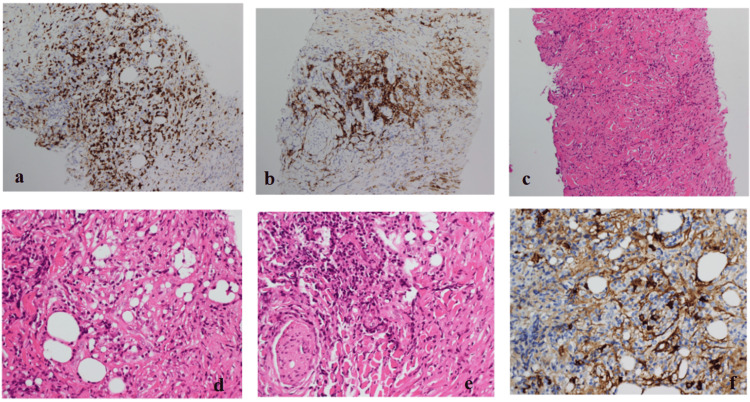
Histopathological features of IgG4-related retroperitoneal fibrosis. Representative histopathological sections from the CT-guided biopsy of the retroperitoneal mass. (a) CD3 immunohistochemical staining demonstrating prominent reactive T-cell–rich inflammatory infiltrates. (b) CD38 immunohistochemical staining highlighting dense plasma cell infiltrates and aggregates. (c) Hematoxylin and eosin–stained section showing adipose tissue entrapped within a fibroinflammatory collagenous lesion, with mixed chronic inflammatory infiltrates including plasma cells and eosinophils. (d) Hematoxylin and eosin–stained section demonstrating an entrapped peripheral nerve surrounded by dense fibrocollagenous stroma and chronic inflammatory infiltrates containing plasma cells and eosinophils. (e) IgG4 immunohistochemical staining showing 14-16 IgG4-positive plasma cells per one high-power field within the inflammatory infiltrate. IgG showed a diffuse marked overstaining, precluding counting of IgG plasma cells and estimating IgG4 to IgG ratio.

During this admission, the patient was empirically treated with a 10-day course of ciprofloxacin for elevated inflammatory markers, although blood and urine cultures remained negative. A rheumatology consultation was obtained in light of the histopathological findings consistent with IgG4-related disease. Review of biopsy specimens (Figure 4) supported the diagnosis. Rheumatology assessment on 11 August 2025 noted worsening renal function and elevated inflammatory markers; however, given concern for a concurrent infection, initiation of definitive immunosuppressive therapy was deferred.

**Figure 5:**
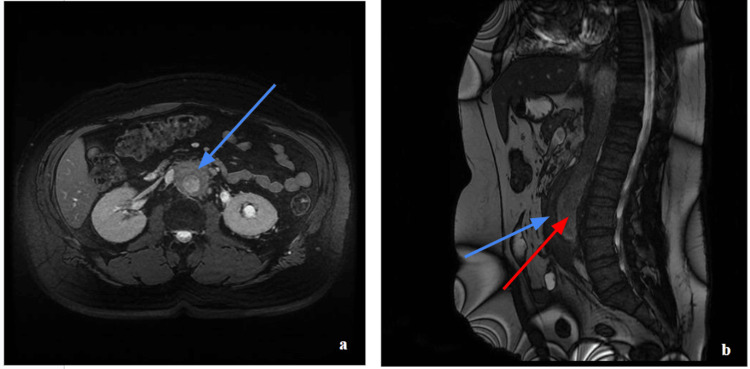
Abdominal MRI demonstrating retroperitoneal fibrosis with associated distal abdominal aortic aneurysm. Axial (a) and sagittal (b) abdominal MRI images demonstrating confluent retroperitoneal soft-tissue thickening surrounding the abdominal aorta and ureters (blue arrows), in keeping with retroperitoneal fibrosis. A fusiform aneurysm of the distal abdominal aorta is also noted (red arrow).

On 13 August 2025, the patient developed shivering and was managed with hydrocortisone and ongoing antibiotic therapy. Autoimmune serological testing, including ANA, ANCA, and complement levels, was unremarkable. Serum IgG4 levels were within the normal range. An MRI of the abdomen reaffirmed the presence of retroperitoneal fibrosis and bilateral ureteric stents and additionally demonstrated a fusiform aneurysm of the distal abdominal aorta (Figure 5).

Following improvement in inflammatory markers and completion of antibiotic therapy, the rheumatology team confirmed the diagnosis of IgG4-related retroperitoneal fibrosis and proceeded with immunosuppressive treatment. The patient received intravenous methylprednisolone pulses for three days, followed by the first dose of rituximab. At discharge, the plan was to continue oral prednisolone at 60 mg daily for four weeks with gradual tapering and to administer a second dose of rituximab two weeks after the initial infusion.

## Discussion

This case illustrates both the typical presentation and the diagnostic and therapeutic complexities associated with immunoglobulin G4-related retroperitoneal fibrosis (IgG4-related RPF) [1,2]. Obstructive uropathy resulting from ureteric encasement by fibroinflammatory tissue is a well-recognized and potentially life-threatening complication of RPF and remains one of the most common causes of acute kidney injury (AKI) in this condition [6]. In the present case, the patient’s initial severe AKI was directly attributable to bilateral ureteric obstruction, with prompt bilateral ureteric stent insertion resulting in rapid and sustained improvement in renal function. This response underscores the critical role of early urinary tract decompression in preventing irreversible renal damage [9,13].

Although serum IgG4 levels were within the normal range, this finding does not exclude IgG4-related disease. Serum IgG4 elevation is reported in approximately 70-80% of affected patients, and normal levels are well described, particularly in organ-limited disease [1]. In this case, the diagnosis of IgG4-related RPF was supported by histopathological findings from a CT-guided biopsy demonstrating a fibroinflammatory spindle cell lesion with IgG4-positive plasma cells, in conjunction with characteristic radiological features of a retroperitoneal mass encasing the major vessels and ureters [3,5,7]. The identification of a fusiform aneurysm of the distal abdominal aorta on MRI further strengthened the diagnosis, as IgG4-related aortitis and peri-aortitis are recognized manifestations of systemic IgG4-related disease [4]. Alternative differential diagnoses, including systemic vasculitis, infection, and drug-induced fibrosis, were systematically excluded through clinical assessment, serological investigations, and histopathological evaluation.

To further support the diagnosis, the patient’s clinical, radiological, and histopathological findings were evaluated against the 2019 American College of Rheumatology/European League Against Rheumatism classification criteria for IgG4-related disease (Table 2) [15]. Despite normal serum IgG4 levels, the cumulative score exceeded the classification threshold, reinforcing the diagnosis in this case.

**Table 2:** Tabular comparison of patient findings with the 2019 American College of Rheumatology / European League Against Rheumatism classification criteria for IgG4-related disease. This table summarizes the patient’s clinical, radiological, and histopathological findings in relation to the 2019 ACR/EULAR classification criteria for IgG4-related disease. The cumulative score exceeded the required threshold for classification despite normal serum IgG4 levels, supporting the diagnosis of IgG4-related retroperitoneal fibrosis. No exclusion criteria were identified.

ACR/EULAR Criterion	Patient findings	Points	Comments
Entry Criteria: Characteristic organ involvement	Retroperitoneal fibrosis encasing aorta and ureters with hydronephrosis	Entry criteria achieved	
Serum IgG4 level	0.70 g/L (within normal range)	0	Does not contribute to score
Histopathology: Dense lymphoplasmacytic infiltrate	Prominent plasma cell–rich inflammatory infiltrate on CD38 staining	+4	Characteristic feature
Histopathology: Storiform fibrosis	Fibroinflammatory spindle cell lesion with dense collagenous stroma	+4	Consistent with IgG4-RD
Histopathology: Obliterative phlebitis	Not reported	0	No clear evidence
Immunostaining: IgG4+ plasma cells	Scattered IgG4-positive plasma cells	+7	Supports diagnosis despite normal serum IgG4
Imaging features	Retroperitoneal soft tissue encasing vessels and ureters with hydronephrosis	+8	Typical RPF pattern
Other organ involvement	Abdominal aortic aneurysm	+4	Supports systemic disease
Exclusion criteria	No malignancy, infection, or alternative diagnosis identified	0	Required condition met

The patient’s subsequent clinical deterioration with a second episode of severe AKI represented a pivotal point in his disease course. IgG4-related disease is characteristically relapsing-remitting, and recurrence of obstructive complications is well described [10]. However, in this instance, the recurrence of renal dysfunction posed a diagnostic challenge. Despite persistently elevated inflammatory markers, multidisciplinary evaluation, particularly from the rheumatology team, raised concern for a concurrent infectious process rather than an IgG4-RD flare. This distinction was critical, as premature initiation of high-dose immunosuppression in the presence of active infection carries significant risk, particularly in patients with advanced renal dysfunction [14]. The decision to defer definitive immunosuppressive therapy until infection had been adequately treated highlights the importance of careful clinical judgment and close collaboration between rheumatology, urology, nephrology, and infectious disease teams.

Following resolution of suspected infection, the initiation of pulse corticosteroid therapy followed by B-cell-depleting treatment with rituximab was consistent with current treatment strategies for severe or organ-threatening IgG4-related disease [11,12]. Rituximab has emerged as an effective option in patients with relapsing disease or in whom prolonged corticosteroid exposure is undesirable, particularly in the setting of significant renal involvement. 

## Conclusions

Finally, this case emphasizes the systemic nature of IgG4-related disease and the need for comprehensive evaluation beyond the primary site of involvement, as reflected by the patient’s extra-retroperitoneal manifestations. The fluctuating renal function observed during recovery further underscores the relapsing course of the disease and the necessity for close, long-term follow-up in specialized multidisciplinary clinics. Overall, this case highlights the importance of timely recognition of obstructive complications, integration of histopathological and radiological findings for diagnosis, and a cautious, evidence-based approach to immunosuppressive therapy in the management of IgG4-related retroperitoneal fibrosis.

## References

[1] Lian L, Wang C, Tian JL (2016). IgG4-related retroperitoneal fibrosis: a newly characterized disease. Int J Rheum Dis.

[2] Rossi GM, Rocco R, Accorsi Buttini E (2017). Idiopathic retroperitoneal fibrosis and its overlap with IgG4-related disease. Intern Emerg Med.

[3] Hu JQ, Jin ZY, Yu YY (2025). Clinical characteristics of IgG4-related retroperitoneal fibrosis in a cohort of 117 patients with idiopathic retroperitoneal fibrosis: a retrospective study. Clin Rheumatol.

[4] Stone JR (2011). Aortitis, periaortitis, and retroperitoneal fibrosis, as manifestations of IgG4-related systemic disease. Curr Opin Rheumatol.

[5] Fujimori N, Ito T, Igarashi H (2013). Retroperitoneal fibrosis associated with immunoglobulin G4-related disease. World J Gastroenterol.

[6] Choi YK, Yang JH, Ahn SY (2019). Retroperitoneal fibrosis in the era of immunoglobulin G4-related disease. Kidney Res Clin Pract.

[7] Liu Y, Zhu L, Wang Z (2021). Clinical features of IgG4-related retroperitoneal fibrosis among 407 patients with IgG4-related disease: a retrospective study. Rheumatology (Oxford).

[8] Mathew B, Gupta D, Gahlot GP, Takkar P (2025). Rare case of IgG4-related retroperitoneal fibrosis mimicking renal cell carcinoma: A diagnostic challenge. Indian J Pathol Microbiol.

[9] Farook S, Jilani MS, Islam MK (2023). IgG4-related retroperitoneal fibrosis: a case report of a challenging disease. Clin Case Rep.

[10] Wang K, Wang Z, Zeng Q (2021). Clinical characteristics of IgG4-related retroperitoneal fibrosis versus idiopathic retroperitoneal fibrosis. PLoS One.

[11] Kim YJ, Kim GE, Ma SK (2020). Case report: IgG4-related renal disease co-existing with retroperitoneal fibrosis. Transl Androl Urol.

[12] Tsai HC, Liao HT, Tsai CY (2021). Retroperitoneal fibrosis with a damaged kidney in IgG4-related disease. J Clin Rheumatol.

[13] Chandna A, Sharma AP, Pareek T (2019). IgG4-related retroperitoneal fibrosis: an emerging masquerader with a sinister presentation. Urology.

[14] Soriano Rios A, Paredes H, Hernández-Calleros J (2019). Retroperitoneal fibrosis. Steroid treatment response seems to depend on its association to IgG4 related disease. Med Hypotheses.

[15] Wallace ZS, Naden RP, Chari S (2020). The 2019 American College of Rheumatology/European League Against Rheumatism classification criteria for IgG4-related disease. Ann Rheum Dis.

